# Alphaviruses Detected in Mosquitoes in the North-Eastern Regions of South Africa, 2014 to 2018

**DOI:** 10.3390/v15020414

**Published:** 2023-02-01

**Authors:** Milehna M. Guarido, Isabel Fourie, Kgothatso Meno, Adriano Mendes, Megan A. Riddin, Caitlin MacIntyre, Sontaga Manyana, Todd Johnson, Maarten Schrama, Erin E. Gorsich, Basil D. Brooke, Antonio Paulo G. Almeida, Marietjie Venter

**Affiliations:** 1 Zoonotic Arbo- and Respiratory Virus Program, Centre for Viral Zoonoses, Faculty of Health Sciences, University of Pretoria, Pretoria 0031, South Africa; 2 Department of Production Animal Studies, Faculty of Veterinary Science, University of Pretoria, Pretoria 0031, South Africa; 3 UP Institute for Sustainable Malaria Control (UP ISMC), Faculty of Health Sciences, University of Pretoria, Pretoria 0007, South Africa; 4 National Health Laboratory Service, Department of Virology, School of Laboratory Medicine and Medical Sciences, University of KwaZulu-Natal, Durban 4041, South Africa; 5 Department of Biological Sciences, Copperbelt University, Kitwe 21692, Zambia; 6 Institute of Environmental Sciences, Leiden University, 2333 CC Leiden, The Netherlands; 7 School of Life Sciences, University of Warwick, Coventry CV4 7AL, UK; 8 The Zeeman Institute for Systems Biology & Infectious Disease Epidemiology Research, University of Warwick, Coventry CV4 7AL, UK; 9 Centre for Emerging Zoonotic & Parasitic Diseases, National Institute for Communicable Diseases/NHLS, Johannesburg 2192, South Africa; 10 Wits Research Institute for Malaria, School of Pathology, University of the Witwatersrand, Johannesburg 2000, South Africa; 11 Institute of Hygiene and Tropical Medicine (IHMTNOVA), Medical Parasitology Unit/GHTM, NOVA University of Lisbon, 1349-008 Lisbon, Portugal

**Keywords:** alphaviruses, Middelburg, Sindbis, Ndumu, mosquitoes, *Aedes*, *Culex*, Africa region

## Abstract

The prevalence and distribution of African alphaviruses such as chikungunya have increased in recent years. Therefore, a better understanding of the local distribution of alphaviruses in vectors across the African continent is important. Here, entomological surveillance was performed from 2014 to 2018 at selected sites in north-eastern parts of South Africa where alphaviruses have been identified during outbreaks in humans and animals in the past. Mosquitoes were collected using a net, CDC-light, and BG-traps. An alphavirus genus-specific nested RT-PCR was used for screening, and positive pools were confirmed by sequencing and phylogenetic analysis. We collected 64,603 mosquitoes from 11 genera, of which 39,035 females were tested. Overall, 1462 mosquito pools were tested, of which 21 were positive for alphaviruses. Sindbis (61.9%, N = 13) and Middelburg (28.6%, N = 6) viruses were the most prevalent. Ndumu virus was detected in two pools (9.5%, N = 2). No chikungunya positive pools were identified. Arboviral activity was concentrated in peri-urban, rural, and conservation areas. A range of Culicidae species, including *Culex univittatus*, *Cx. pipiens* s.l., *Aedes durbanensis*, and the *Ae. dentatus* group, were identified as potential vectors. These findings confirm the active circulation and distribution of alphaviruses in regions where human or animal infections were identified in South Africa.

## 1. Introduction

The alphavirus genus (Family Togaviridae) contains a diverse group of viruses, associated with disease in humans and animals. For the most part, alphaviruses can be separated according to geographic distribution [1]. The Old-World group, including the Chikungunya (CHIKV), O’nyong–nyong (ONNV), Ross River (RRV), Semliki Forest (SFV), and Sindbis (SINV) viruses, is mostly associated with rash and arthritis [2]. The New World group includes eastern equine encephalitis virus (EEEV), Venezuelan equine encephalitis virus (VEEV), and western equine encephalitis virus (WEEV), are associated with neurological complications and encephalitis in horses and humans in the Americas [3]. For some of the African alphaviruses, such as Middelburg (MIDV) and Ndumu (NDUV) viruses, the vectors are not well known.

As arboviruses, alphaviruses replicate in both invertebrate hosts (mostly mosquitoes) and vertebrate hosts (e.g., birds, equids, amphibians, reptiles, rodents, swine, humans, and other primates) [4]. *Aedes* and *Culex* mosquitoes are the main vectors of alphaviruses. *Aedes aegypti* and *Ae. albopictus* are associated with CHIKV outbreaks in Africa and with expansion to the Reunion Islands, Europe, Asia, and the Americas [5], while *Culex* spp. have been identified as the main vectors of SINV in Europe [6] and historically in South Africa [2,7]. However, other genera of mosquitoes have also been found to be involved in their transmission, including *Anopheles*, *Culiseta*, and *Mansonia* mosquitoes [8].

Alphaviruses have been largely neglected in Southern Africa in recent years. Chikungunya virus outbreaks are infrequent in South Africa, and have been previously reported in rural tropical wooded savannah of the eastern Limpopo, and Northern KwaZulu-Natal provinces in the 1970’s. The primary vector is *Ae. furcifer* and possibly also *Ae. cordellieri* [9,10,11]. Sindbis virus is the most common alphavirus associated with human disease in the region, with cases identified annually in South Africa, where large outbreaks were reported in the 1970′s and 1980′s [2]. More recently, MIDV (in 2017) and SINV (2019–2020) cases were detected from hospitalized patient samples by PCR and serological methods in two northern provinces of South Africa [12,13]. Febrile and neurological cases have also been described in horses in recent years [14]. MIDV co-infections with other viruses such as West Nile virus, SINV, and African horse sickness virus (AHSV) have been associated with fatalities in horses, indicating that MIDV could contribute to a more severe clinical outcome for other infections [12,14]. The amplifying hosts are mainly migratory birds and ornithophilic mosquitoes, specifically *Culex* spp. This mosquito genus is considered to include the main vectors in South Africa [6], namely *Cx. univittatus*, considered the primary vector on the temperate inland plateau, with *Cx. neavei* playing a role in the KwaZulu-Natal lowlands [11]. The Middelburg virus was originally isolated in South Africa in 1957. Two isolates were reported from *Ae. caballus* and *Aedes* spp. mosquitoes [15]. The vectors for MIDV are not well characterized, but the virus has been detected in entomological surveys in South Africa between 1957 and 1970 in *Ae. caballus*, *Ae. circumluteolus*, *Ae. albocephalus*, and *Ma. africana* [15,16,17,18]. Middelburg virus was later isolated from the spleen of a horse in Zimbabwe that presented with severe clinical disease [19]. Sindbis virus and MIDV have recently been associated with neurological syndromes in horses, livestock, and wildlife in southern Africa [14,20]. Few other alphaviruses have been reported in recent years in South Africa.

The aim of the study was to obtain current data regarding alphaviruses circulating in the north-eastern provinces of South Africa, where most neurological cases were identified in humans and animals, and identify the potential associated vectors.

## 2. Materials and Methods

### 2.1. Study Areas

**Figure 1:**
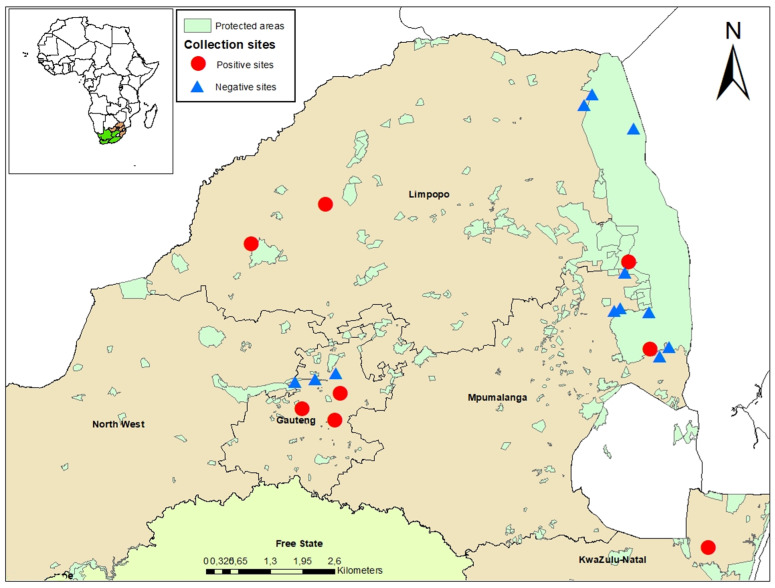
Mosquito collection sites indicating collection locations for alphavirus-positive (circles) and negative (triangles) mosquito pools. Sites were surveyed from January 2014 to May 2018 in South Africa.

The study was conducted in five provinces of South Africa, including Gauteng, Limpopo, the Northwest Provence, Mpumalanga, and KwaZulu-Natal (Figure 1) [21]. Prior to the start of the current study in 2014, a pilot study was conducted in 2011 where mosquitoes were collected, which helped to select and establish the sentinel sites based on historical arboviral outbreaks in livestock and/or wildlife. Mosquitoes were sampled monthly from January 2014 to May 2017 from sentinel sites. Supplementary collections were made in ad-hoc sites, following detection of arboviral occurrence in animal or human hosts. During 2018, only one collection per site was performed from January to May. Additional collection was performed from March to April 2017 in and around Kruger National Park (KNP) [22].

Sentinel site mosquito capture in Gauteng Province was performed in peri-urban areas on horse farms (Boschkop and Kyalami) [21]. In Limpopo Province, sampling was performed in conservation areas (Marakele National Park and Lapalala Wilderness game reserve) [19]. Ad-hoc collections were made in Gauteng Province, Limpopo Province, Northwest Province, KwaZulu Natal Province, and Mpumalanga Province across three different land use types: urban, peri-urban, rural, and conservation areas [21].

The sampling period and number of traps varied across sites depending on the property’s size and access. Trapping was carried out from 15:30–16:00 to 5:00–8:00 and was conducted for 1–3 consecutive nights per site, using multiple types of carbon dioxide (CO_2_)-baited traps: net, CDC miniature light (BioQuip Products, Rancho Dominguez, CA, USA), and BG-Sentinel (BioGents Corporation, Regensburg, Germany). Mosquitoes were collected from the net traps using hand-held aspirators and transferred to mesh-topped polystyrene cups. The CDC miniature light traps were hung at least 1.5 m from the ground and baited with CO_2_ and multiple lights of differing wavelengths (incandescent, LED, UV white, and UV black). BG-Sentinel traps were added in 2017 and baited with a non-toxic lure and CO_2_. All the traps were placed at least 80 m apart and out of line of sight from each other to ensure no interference occurred.

Collected mosquitoes were immediately euthanized by freezing and were morphologically identified to species using published keys and descriptions [23,24,25,26]. Females were pooled (predominantly ≤50 individuals per pool) by species, site, and month and preserved at −80 °C until testing. Mosquitoes sampled from January to June were selected for screening. The freshly engorged mosquitoes were not tested for virus screening and were separated for blood meal analyses, more details can be seen in Guarido et al. (2021) [21]. Climatic data were obtained from the South African Weather Service.

### 2.2. Mosquito Processing

The mosquito pool homogenization was carried out in a biosafety level 3 (BSL3) laboratory based at the University of Pretoria, Centre for Viral Zoonoses (CVZ). Briefly, to perform the homogenization, five sterile glass beads were placed in microcentrifuge tubes (Eppendorf, Hamburg, Germany) containing 2 mL of reconstituted minimum essential medium (Gibco, Life Technologies, Carlsbad, CA, USA), and the mosquitoes from each pool were shaken vigorously in a TissueLyser. The homogenates were clarified by centrifugation at 3000× *g* for 30 min at 4 °C and stored at −80 °C. Viral RNA was extracted from 200 µL homogenate using the RNeasy Mini Kit (Qiagen, Valencia, CA, USA) according to the manufacturer’s instructions.

### 2.3. Detection and Analysis by RT-PCR

Extracted viral RNA was screened for alphaviruses by targeting a 481 bp region of the non-structural protein 4 (nsP4) gene using published generic genus primers [27] and the SuperScript III One-Step RT-PCR System with Platinum Taq DNA polymerase (Thermo Fisher Scientific, Waltham, MA, USA). To distinguish between MIDV and SINV positives, an alphavirus genus-specific nested real-time PCR was performed using TaqMan probes (Roche Life Science, Mannheim, Germany) on the Roche LightCycler 2.0 with MIDV and SINV specific probes as previously published, resulting in a 198 bp PCR product [14].

In order to improve the phylogenetic analysis, a nested PCR amplifying a larger 351 bp fragment of the nsP4 gene was used for MIDV and SINV positive samples, using previously optimized MIDV and SINV specific primers [20]. In addition, a primer set targeting the glycoprotein E of MIDV and SINV was used to improve phylogenetic distinction [14,27]. For MIDV, the E segment could also help to detect recombination events since full genome sequencing of the virus has suggested that MIDV EI was generated by recombination with a Semliki-like virus [19]. For MIDV, a nested PCR was performed in a volume of 25 µL including 5 µL of the first-round product and Platinum Taq DNA polymerase (Invitrogen, California, USA). For SINV, a nested PCR using the same method used for MIDV with the exception that the final reaction was 23 µL with 2 µL of the first-round product, using A1 (10 pmol) and A2 (10 pmol) primers [28] were performed. For the Ndumu virus, a primer set targeting the glycoprotein E1 was utilized as previously described [29]. All the primers used in the alphavirus detections are shown at Table S1.

### 2.4. Virus Isolation

All virus isolations were attempted in the BSL-3 facility at the University of Pretoria, CVZ. Briefly, virus isolation was attempted from all positive pools detected using their homogenate on African green monkey kidney cells (Vero) and baby hamster kidney cells (BHK-21) clone 3 (BSR) cells. Cells were grown in 25 cm^2^ flasks maintained in Eagle’s Minimum Essential Medium (EMEM, Sigma Aldrich, St. Louis, MO, USA), supplemented with 10% (*v*/*v*) fetal Bovine Serum (FBS) for Vero cells or 20% (*v*/*v*) for BSR cells (FBS, Sigma Aldrich), and 1X (*v*/*v*) MycoZap Plus-CL (LONZA, Basel, Switzerland) at 37 °C and 5% CO_2_ until a monolayer of ~80% was obtained. For infection, growth media were discarded and cells were washed three times with PBS (Phosphate Buffered Saline, Sigma Aldrich); 200 µL of mosquito homogenate were allowed to adsorb for 1 h at 37 °C with 5% CO_2_ followed by the addition of 5 mL of EMEM, supplemented with 2% FBS and MycoZap Plus-CL. Cells were monitored for CPE for between 7 and 10 days. Subsequent passaging was performed by inoculating new cells using 500 µL of supernatant from the previous passage, following 3× freeze thawing at −80 °C, and clarification by centrifugation. A 140 µL aliquot of supernatant from cultures was used for viral RNA extraction, and alphaviral RNA was confirmed by the nested real-time PCR as described above.

### 2.5. Whole Genome Sequencing Using Illumina iSeq

Culture isolates were extracted in order to obtain full genome sequences for virus characterization. Briefly, RT-PCR confirmed virus cultures were grown in three T75 flasks per sample, combined, and spun at 1008× *g* per 30 min to remove cell debris. After which the supernatant was filtered and concentrated with Millipore Amicon Ultra-15 centrifugal filter units 10K (Merck, Rahway, NJ, USA) by centrifugation at 5000× *g* for 30 min. The concentrated culture was used for RNA extraction using the QIAmp Viral RNA Mini Kit (Qiagen, Hilden, Germany) according to the manufacturer’s instructions. Double-stranded cDNA synthesis was performed on the extracted RNA using the Maxima H-minus double-stranded cDNA Kit (Thermo Fisher Scientific) according to the manufacturers’ instructions. The cDNA was purified using the DNA Clean and Concentrator™—5 Kit according to the manufacturer’s instructions. Following clean up, each sample was eluted in 12 μL elution buffer, and the concentration of each sample was measured on the Qubit fluorometer (Thermo Fisher Scientific). The library preparation was performed as per the Nextera DNA Flex Library Preparation Kit (Illumina, San Diego, CA, USA) protocol. The pooled library was loaded onto the Illumina^®^ iSeq™ 100 system according to the Illumina sequencing guide (Illumina) for NGS.

### 2.6. Molecular Identification of Mosquito Samples

In order to confirm the morphological identification to species level where possible, DNA was extracted from 50 µL of the mosquito homogenate using DNeasy Blood & Tissue Kit (Qiagen, Valencia, CA, USA), according to the manufacturer’s instructions. The DNA barcode region of the mitochondrial DNA of subunit I of the cytochrome oxidase (COI) gene was amplified using universal primers [30].

### 2.7. Gel Electrophoresis and Sequencing

PCR products were viewed on a 2.0% agarose gel containing ethidium bromide following electrophoresis. Amplicons of the correct size were excised from the gel and purified using a Zymoclean Gel DNA Recovery Kit (Zymo Research, Irvine, CA, USA) according to the manufacturer’s instructions. Purified amplicons were bidirectionally sequenced using the BigDye Direct Sanger Sequencing Kit (Thermo Fisher Scientific, Waltham, MA, USA) and sent to the University of Pretoria DNA sequencing facility or Inqaba Biotec (Pretoria, South Africa) for Sanger sequencing.

### 2.8. Data Analysis and Phylogenetic Analysis

Resulting sequences were edited and analyzed in CLC Main Workbench version 8.0.1 [31]. Viral sequences were compared to the databases available in the GenBank [32], using the Basic Local Alignment Search Tool (BLAST) [33]. For COI, representative mosquito sequences were selected on the GenBank and BOLD [34] databases. Multiple sequence alignments were compiled using the online version of Multiple Alignment using Fast Fourier Transform [35] with default parameters. A model of best fit was identified, and a maximum likelihood tree was produced using Molecular Evolutionary Genetics Analysis (MEGA) version 7.0 [36].

FASTQ files were removed from the Illumina^®^ iSeq™ 100 system and analyzed using the Qiagen CLC Genomics workbench (Qiagen, Valencia, CA, USA). This included assembly for the SINV isolate S.A.AR86 and the reference genome (U38305.1). Consensus sequences were combined with full genome sequences from GenBank and aligned using the online version of MAFFT version 7 [35] using default parameters. Sequences for alignment and phylogenetic assessment were selected according to the gene segment and strain and downloaded in a FASTA format. The aligned sequences were viewed and edited in Molecular Evolutionary Genetics Analysis version 7.0 (MEGA 7). Model test analysis was conducted with the default settings using jModeltest. The appropriate dataset was specified, and a maximum likelihood tree with a bootstrap value of 1000 replicates was generated using Maximum Likelihood (ML) and/or Bayesian Inference method using Bayesian evolutionary analysis by sampling trees (BEAST).

Graphics were created in Microsoft Excel (Version 2010) [37]. Minimum infection rates of mosquito infections were calculated per positive species detected: ([number of positive pools/total specimens tested] × 1000). The infection rate was calculated based on the assumption that a positive pool contains at least one infected mosquito and considering the unequal pool size.

## 3. Results

### 3.1. Mosquito Collection

A total of 64,603 adult mosquitoes belonging to 11 genera were collected across four sentinel sites and *ad-hoc* sites. The most common genus collected was *Culex* (38.9%, N = 25,131) followed by *Anopheles* (33.3%, N = 21,494), *Aedes* (18.6%, N = 12,037), *Mansonia* (6.2%, N = 3987), and other genera combined (3.0%, N = 1954, *Uranotaenia*, *Aedeomyia*, *Ficalbia*, *Coquillettidia*, *Mimomyia*, *Culiseta*, and *Eretmapodites*).

In Marakele (Limpopo Province), *Cx. poicilipes* was the most abundant species (34.4%), followed by *Ae. mcintoshi* (20.9%). In Lapalala (Limpopo province), the most abundant species was *Anopheles coustani* (20.5%), followed by *An. theileri* (16.2%). In Gauteng province, Boschkop had an abundance of *Cx. univittatus* (43.5%), followed by *Cx. theileri* (19.5%), and in Kyalami *Cx. theileri* (34.8%) was the most abundant, followed by *Cx. pipiens* s.l. (19.1%). At the *ad-hoc* sites where alphavirus cases were detected, Mnisi (Mpumalanga province) had an abundance of *Cx. poicilipes* (35.5%) followed by *Ma. uniformis* (18.6%), while in Benoni (Gauteng province) *Cx. univittatus* (25.3%) was the most abundant species followed by *Ae. dentatus* group (19.6%), and in Hectorspruit (Mpumalanga) *Cx. pipiens* s.l. (46.6%) was the most abundant species followed by *Ae. aegypti* (15.4%). Further information about the collection data and abundance of species is available in Guarido et al. [21].

**Figure 2:**
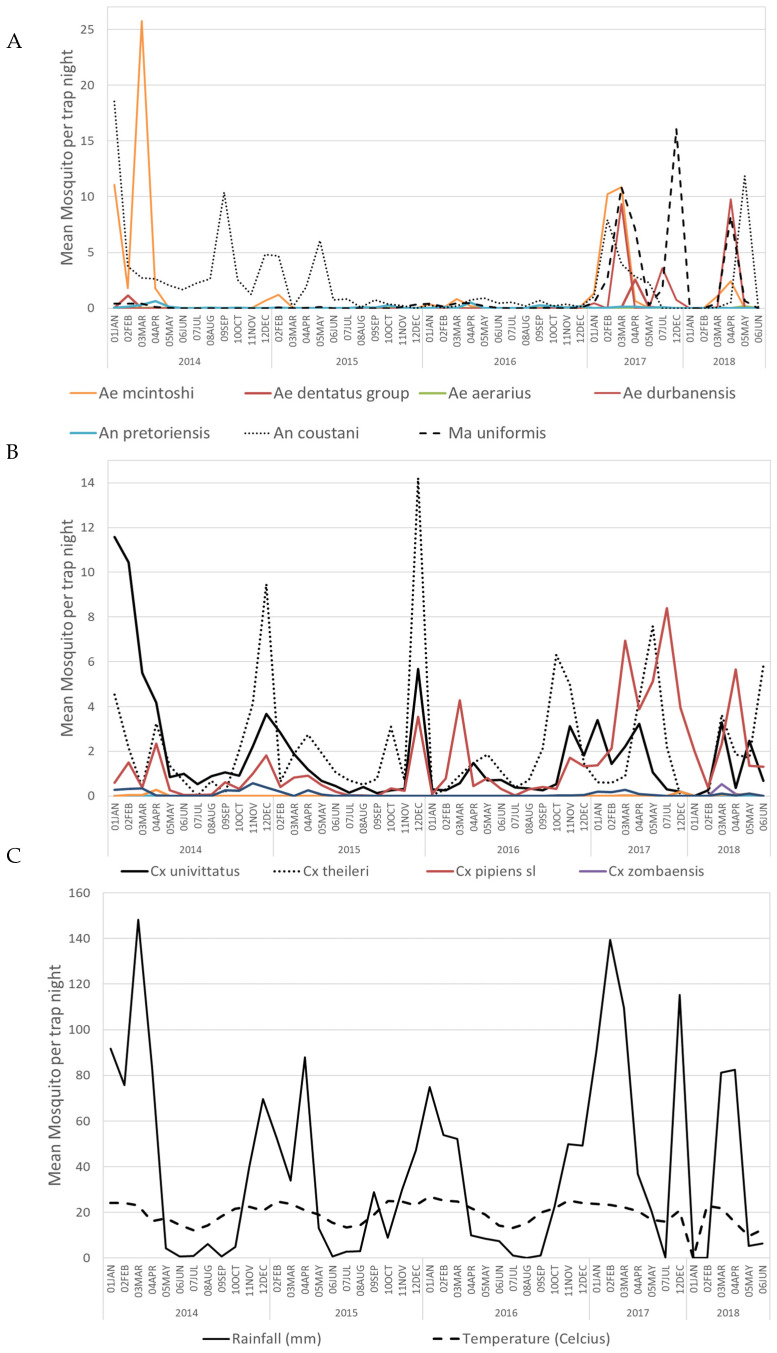
Abundance per trap/night of alphavirus-positive mosquito species. (**A**) Positive *Culex* species abundance per trap night, (**B**) Positive *Aedes*, *Anopheles*, and *Mansonia* species per trap night; (**C**) Rainfall and temperature from January 2014 to May 2018.

The average collection per trap/night at the sites fluctuated during the study period. An overall trend was evident, with mosquito densities (mean number of mosquitoes per trap-night) being lower in the drier months, usually from June to October, than during the wetter months (October/November–April/May). The mosquito populations peaked with the heavy rains that coincided with the highest mean temperatures. We also noted that broader rainfall periods, as in between 2016 and 2017, resulted in broader mosquito capture, for example multiple *Culex* and *Aedes* species. (Figure 2).

### 3.2. Alphavirus Detection

A total of 21 of the 1462 mosquito pools tested (1.4%) were found to be positive for alphavirus RNA (Table 1). These included 13 pools positive for SINV RNA, six pools positive for MIDV RNA, and two pools positive for Ndumu virus RNA, representing 61.9, 28.6, and 9.5% of the 21 alphavirus-positive pools for SINV, MIDV, and NDUV, respectively (Table S2). Alphaviruses were detected in mosquitoes from all sentinel sites (Marakele, Lapalala, Kyalami, and Boschkop) and at the ad-hoc sites: Benoni (Gauteng), Mnisi (Mpumalanga), Jozini (KwaZulu Natal province), and Hectorspruit (Mpumalanga).

As shown in Table 1, mosquitoes belonging to the *Culex*, *Aedes*, *Anopheles*, and *Mansonia* genera tested positive for alphaviruses. *Culex univittatus* showed five positives for SINV and one for MIDV, but the infection rate was low due to the large sample size. *Aedes* mosquitoes showed a higher species variety that tested positive, including *Ae. durbanensis*, *Ae. dentatus* group, *Ae. aerarius*/*tarsalis*, and *Ae. mcintoshi*.

During the study period, Jozini (Northern KwaZulu-Natal Province) samples produced the greatest variety of alphaviruses detected (SINV, MIDV, and NDUV). SINV detections were slightly higher in the peri-urban areas (53.3% of the total SINV positive pools), followed by rural areas (26.7%), and conservation sites (20%). MIDV detections were equally prevalent in peri-urban sites and rural areas (42.9% for each habitat out of the total positive MIDV detected) and higher than found in conservation sites (14.3%). Ndumu virus occurred in two pools in Jozini (rural) and one pool in Lapalala (conservation).

**Figure 3:**
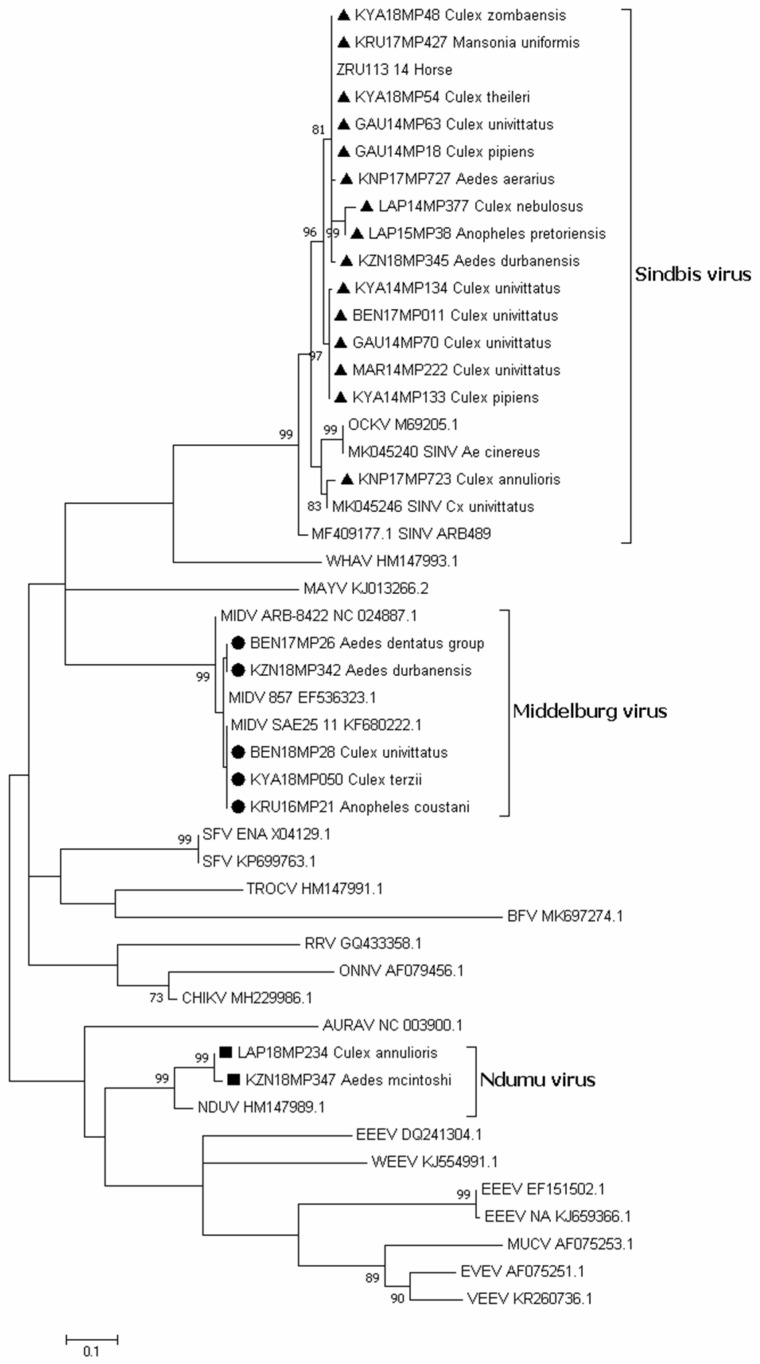
Phylogenetic comparison of the gene nsP4 alphavirus-positive sequences identified in this study. The phylogenetic tree of the positive sequences is based on the 48 sequences of the 180 bp of the nsP4 gene used for alphavirus genus PCR identification. The tree was constructed by employing the program MEGA 7 and the maximum likelihood method based on the Kimura 2-parameter model with 1000 bootstrap replicates. GenBank accession numbers are indicated. Numbers on internal branches indicate bootstrap values. The samples that are a part of this study are marked with a triangle (SINV), a dot (MIDV), and a square (NDUV).

**Figure 4:**
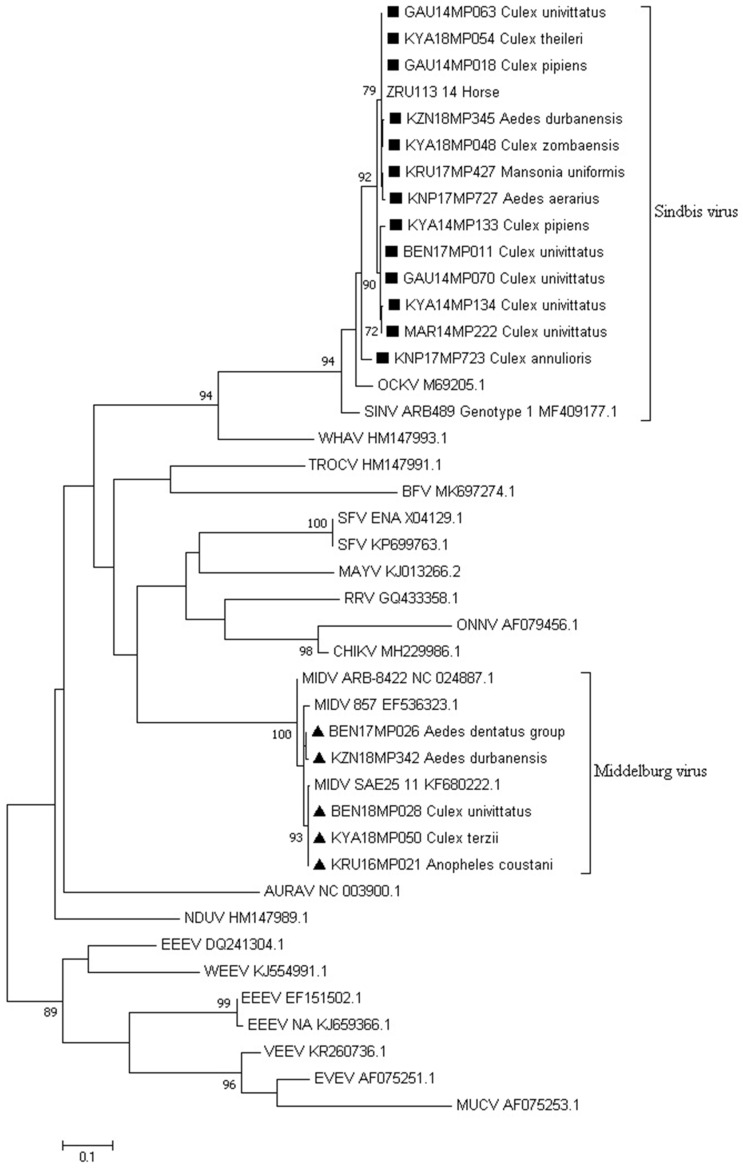
Phylogenetic comparison of the MIDV and SINV positive sequences identified in this study. The tree was constructed relative to 42 reference sequences of a 481 bp region of the nsP4 gene by employing the program MEGA 7 and the maximum likelihood method based on the Tamura3-parameter model and 1000 bootstrap replicates. GenBank accession numbers are indicated. Numbers on internal branches indicate bootstrap values. The samples that are a part of this study are marked with a square (SINV) and a triangle (MIDV), and the mosquito species they were identified as is indicated.

Sequencing of alphavirus positive pools (Table S2) followed by maximum likelihood phylogeny based on a small fragment (180 bp) of the nsP4 gene confirmed BLAST analysis that all the positive pools detected were either SINV, MIDV, or NDUV (Figure 3). One pool from Jozini (KZN18MP346) tested positive by PCR but could not be sequenced and is not shown in Figure 3. We were able to confirm the positivity of the pool as the E segment was successfully amplified and sequenced (Figure S1). The maximum likelihood phylogeny based on the larger fragment (481 bp) on the nsP4 gene region confirmed that the SINV positive pools detected belonged to genotype 1 and clustered with an isolate from a horse detected in South Africa in 2014 (Figure 4). In both phylogenetic analyses, four out of five SINV isolates from *Cx. univitattus* formed a monophyletic cluster, with one isolate (GAU14MP63) grouping with SINV from other *Culex* species. SINV from *Cx annuloris* clustered separately from all other SINV isolates. All five MIDV positives (Figure 4) were also confirmed as MIDV and clustered with previously positive horse strains isolated from South Africa and Zimbabwe. P-distance analyses demonstrated high nucleotide similarities (97.6% to 100.0%) for MIDV between the sequences analysed for this region of nsP4. For SINV, the nucleotide similarities were also high but lower than MIDV (90.8 to 100.0%) compared to published sequences. For NDUV, the similarities ranged from 95.0% to 99.0% for the 180 bp region of the nsP4 gene from the strains identified in this study and the historical strain. We were unable to amplify a larger region of the nsP4 for NDUV with generic alphavirus primers that could amplify MIDV and SINV. An additional region of the E1 region could be amplified with alphavirus generic primers for MIDV (three pools), and NDUV (one pool) but SINV could not be amplified. To further the phylogenetic analysis, Sindbis virus-specific primers could be used to amplify a region of the E2 gene for five SINV positive pools (Figure S1). Sindbis virus strains identified in mosquitoes in 2018 clustered separately from 2014 isolates for the E2 region.

### 3.3. Virus Isolation

An attempt to culture the homogenates of the positive pools detected was made, but no MIDV and NDUV positive pools showed cytopathic effect (CPE) following four passages in both Vero and BHK cells, a result that was confirmed by negative nested real-time PCR testing. A possible explanation could be that virus isolation was only attempted on Vero cells or BHK cells without prior amplification on insect cells such as C6/36. Four isolates were successfully cultured from positive pools of SINV and showed a cytopathic effect (CPE) following four passages in Vero cells; the pools were from *Cx. univittatus* from Boschkop (GAU14MP070), Marakele (MAR14MP222), Kyalami (KYA14MP134), and *Cx. pipiens* s.l. (KYA14MP133).

### 3.4. Whole Genome Sequencing Using Illumina iSeq

Near full genome sequences of SINV were obtained from the four cultured isolates (GAU14MP070, MAR14MP222, KYA14MP133, KYA14MP134). The genomes were 11,600 nucleotides in length, inclusive of the 5′ untranslated region (UTR) and 3′ polyadenylate tail, with a G + C content of 51.4%. Two open reading frames were identified, 7560 nucleotides that encodes the non-structural polypeptides and one of 3622 nucleotides that encodes the structural proteins. The proteins, nsP1 (1–1624 nt); nsP2 (1625–4045 nt); nsP3 (4046–5671 nt); nsP4 (5672–7501 nt) and structural proteins C (7549–8340); E3 (8341–8532 nt); E2 (8533–9801 nt); 6K/TF (9802–9894 nt); and E1 (9895–11284) were identified and annotated. An opal or read-through termination codon (tag) was identified at 5651–5653.

**Figure 5:**
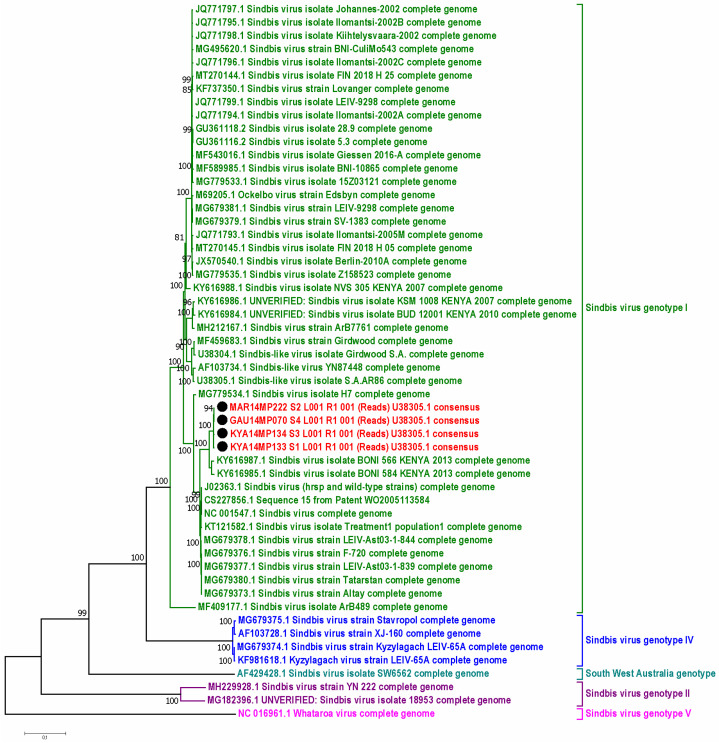
A maximum likelihood phylogenetic tree of the full genome sequences of Sindbis virus (11,592 bp), including the four new sequences generated from mosquito pools from this study (black circles). The evolutionary history was inferred by using the Maximum Likelihood method based on the General Time Reversible model. The solid black circles represent the four mosquitoes from which the SINV full genome was obtained. Red sequences were identified in this study, and they clustered in genotype I with the sequences labeled green. Genotype II is labeled purple, genotype IV navy blue, genotype V pink, and the South-West Australia which clusters on its own is labeled turquoise blue.

By using maximum-likelihood and P-distance analysis, the isolates were compared to 50 SINV complete genomes, with each genotype represented. The four new SINV sequences formed one cluster with a single common ancestor. The phylogeny from whole genome sequencing was therefore similar to the phylogeny achieved from Sanger sequencing. The closest sister group between the new sequences and the reference strains was with a set of two Kenya BONI 2013 strains, all together forming a clear subcluster in genotype I (Figure 5). The P-distance analysis (Table S3) showed that the four isolates had a 99–100% nt and aa similarity between themselves and a 98% to 100% nt and aa similarity to the SINV virus isolate BONI 584/566 (KY616985.1 and KY616987.1) strains, similar to the nsP4 and E protein sequence analysis. The full genome aa sequence p-distance analysis further confirmed that there was a 90–96% aa similarity shared in genotype I and a 96–99% aa similarity in genotype I subtype c. The 96–99% aa similarity in genotype I c accounted for a range of 97–170 aa differences, whereas the 90–96% in the whole genotype accounted for 97–490 aa differences in the complete genome. The differences in the number of aa in the different genotypes as compared to genotype I ranged from 800–1249 (60–80%).

### 3.5. Molecular Identification of Mosquito Samples

DNA barcode sequences consisting of 517 bp were used to build a maximum likelihood tree (Figure S2) as confirmation of morphological mosquito identification. The results show the evolutionary distances using the General Time Reversible model [38] with a bootstrap test with 1000 replicates [39]. Morphological identification of *An. coustani*, *An. pretoriensis*, *Cx. pipiens* s.l., *Cx. duttoni*, *Cx. annulioris*, *Cx. univittatus*, and *Ae. mcintoshi* was confirmed by the COI segment relative to reference sequences on GenBank and BOLD. One pool collected in 2014 in Marakele (pool ID: MAR14MP222), which morphologically was identified as *Cx. univittatus*, showed sequence identity (100%) with *Cx. perexiguus* from Pakistan (GenBank accession KF406802.1) and clustered with *Cx. perexiguus*. The genetic differentiation of a pool collected in 2017 in KNP (pool ID: KNP17MP727), thought to be *Ae. aerarius*, sequenced as *Ae. tarsalis*. Pools morphologically identified as *Ae. dentatus* group and *Ae. durbanensis* could not be confirmed by COI sequences due to the absence or lack of sequences available in the GenBank and Bold databases. This study therefore provides the first *Ae. durbanensis* sequences available from South Africa in GenBank. Four pools (4/23), *Cx. terzii* (pool ID: KYA18MP50), *Cx. univittatus* (KYA14MP134), *Ma. uniformis* (KRU17MP427), and *Cx. annulioris* (LAP18MP234), could not be amplified and, therefore, their morphological identifications could not be confirmed by sequencing.

## 4. Discussion

This study used field surveillance of mosquitoes and PCR screening for members of the alphavirus genus to identify circulating viruses at specific sites in the north-eastern provinces of South Africa. Alphavirus-positive pools were all shown to be SINV, MIDV, or NDUV by sequencing and phylogenetic analysis, and were detected in conservation, rural, and peri-urban areas. None of the alphavirus-positive pools were detected in urban sites, with most being from the peri-urban areas, including several horse properties on the outskirts of major cities such as Pretoria and Johannesburg. This result was not surprising due to the small sample size of mosquitoes collected in urban areas; more details about the collection can be seen in Guarido et al. [21].

The screening period selected represented the arbovirus season in South Africa, when most animal and human cases are detected. In Southern Africa, the summer rainfalls usually occur from November to April [40]. During the same period, the availability of mosquito larval sites peaks due to the hotter and wetter weather conditions. As a result, mosquito abundance increases and peaks from January to mid-April [41]. Therefore, the potential for arboviral transmission and circulation is highest from January to June. The generic alphavirus genus-specific primers described by Sánchez-Seco et al. [27] were able to detect MIDV, SINV, and NDUV in these settings. Although the primers should theoretically detect all members of the alphavirus, due to the high diversity of viruses belonging to the alphavirus genus, the authors were not able to test all viruses and were unable to assay other members from the MIDV and NDUV complexes [27]. Middelburg virus and SINV have previously been detected in horses with acute neurological and febrile illness by using the above described primers in combination with novel probes which can distinguish MIDV and SINV, as these are the most frequently encountered alphaviruses in South Africa [14]. This is the first known successful detection of NDUV using this assay. A positive pool from Kyalami (KYA18MP050) had a small sample size (Table S2), but it was detected positive using two different primer sets. Further analyses are necessary to understand the importance of the detection of such a small sample size, which could be a coincidental finding.

Four SINV-positive mosquito pools could be cultured on Vero cells and showed a cytopathic effect (CPE) following four passages. They were also confirmed by nested RT-PCR. Full genome sequences were obtained from these isolates. An analysis of all the complete genome sequences available showed that there was considerable genetic diversity amongst the strains of SINV that occur worldwide, which can differ in their nucleotide sequences by up to 20%, and this has also been reported previously by Kurkela et al. [42]. However, sequences within the same genotype show greater conservation of 90% or greater, potentially illustrating that particular genotypes evolve in specific niches. Although the number of SINV full genome sequences is still relatively small, sequences isolated from similar locations appear to cluster together. This is exemplified by the two clusters formed by the South African strains described in this study and the sister group of Kenyan strains.

During the study period, SINV was more frequently detected in the mosquito pools than MIDV. Also, it was found to be detected slightly more frequently in peri-urban sites than in conservation and rural areas. Middelburg virus, on the other hand, was equally distributed in peri-urban sites and rural areas in comparison to the conservation sites sampled. Positive pools of mosquitoes detected in peri-urban, rural, and conservation areas could indicate that livestock and wildlife may play an important role in the amplification of these viruses, although it may also reflect areas with greater bird life or more bodies of water conducive for vector mosquito immature development.

Febrile and neurological cases associated with both SINV and MIDV have been described in horses in South Africa. Steyn et al. [20] also demonstrated that alphaviruses, such as MIDV and SINV, might be associated with neurological disease in wildlife, non-equine domestic animals, and birds in South Africa. The Sindbis virus has been shown to use birds as amplifying hosts and has been associated with outbreaks of human disease in South Africa and other regions, including Egypt and Northern Europe [6]. Sindbis virus cases are common in humans in South Africa [2,7,12,13] but less is known about MIDV and NDUV in humans. Serological evidence of MIDV has been demonstrated in humans in South Africa since the discovery of the virus [15]. A MIDV positive pool from the *Ae. dentatus* group was detected during an outbreak of MIDV on a horse farm in a peri-urban site in Benoni, Gauteng Province, where several horse cases were detected during the same season (to be reported elsewhere). The *Ae. dentatus* group was found to be the most abundant mosquito species that occurred during this outbreak on this horse farm. Females of this group are known to feed readily on humans and larger domestic animals [43].

The Ndumu virus was first detected in 1961 from pools of mosquitoes captured in Northern KwaZulu-Natal Province in South Africa [44]. The mosquitoes from which the virus was detected were *Ma. uniformis* and *Ae. circumluteolus* [44]. Although antibodies to the NDUV have been identified in humans, the virus has not been associated with human morbidity yet [44,45]. Little is known about NDUV vertebrate hosts, and in Uganda, domestic pigs were identified as a potential vertebrate host [46]. Here, NDUV was detected in Jozini, a rural area located in KwaZulu-Natal Province, and Lapalala, a conservation area in Limpopo Province. The Jozini site exhibited the greatest variety in alphaviruses. *Aedes durbanensis* was the most abundant aedine in Jozini, and pools of this species were found to be positive for MIDV and SINV. This could suggest that this species may be of epidemiological importance for the region.

To recognize a mosquito species as a vector of an arbovirus, the WHO specified some criteria [47,48] which include: (1) recovery of the virus from wild-caught specimens, (2) demonstration of ability to become infected by feeding on a viraemic host or an artificial substitute, (3) demonstration of ability to transmit biologically by bite, and (4) accumulation of field evidence confirming the significant association of the infected arthropod with the appropriate vertebrate population in which disease or infection is occurring [47,48]. Some species analyzed in this research satisfied some of the WHO criteria and could be considered suspected vectors of MIDV, SINV, and NDUV. *Aedes durbanensis* is an example of a species that could be considered a suspect vector of alphaviruses in the KZN Province because of alphaviruses detected in multiple pools, and they were the most abundant Aedes species collected at the Jozini site. Previously, this species has been shown to feed on livestock animals (cattle, goats, and sheep) [21]. The *Aedes dentatus* group is another group that could play a role as a vector for alphaviruses, particularly in the Highveld region. They were the most abundant species collected in the Highveld in Gauteng Province. This species tested positive for MIDV during an outbreak of MIDV on a horse farm. Previously, horse-derived blood was found in recently engorged individuals [21]. Further studies focusing on vector competence, experiment on species that viruses were detected in, and it would be necessary to clarify their role as vectors, and to satisfy the WHO criteria for describing them as confirmed arboviral vectors.

Sequencing of the barcode COI gene enabled confirmation of most of the morphological mosquito identifications. This was necessary as there is a possibility of morphological misidentification and/or a mixture of species in a pool, which could explain why the pools are not amplified by COI. There is a lack of sequences belonging to African species in the GenBank and Bold databases, and the sequence of *Ae. durbanensis* was the first sequence available in the online databases. The *Cx. univittatus* group consists of species that have external similarities between *Cx. univittatus*, *Cx. neavei*, and *Cx. perexiguus*. According to current knowledge, *Cx. univittatus* is restricted to more Highveld conditions and is replaced by *Cx. neavei* in the warmer Lowveld regions of South Africa [49,50]. It has been established that *Cx. neavei* and *Cx. univittatus* are vectors for SINV, and they have also been considered important vectors for West Nile and Usutu viruses [7,8]. Mixão et al. [51] showed that *Cx. univittatus,* from the type locality in South Africa and from the Iberian Peninsula, are closely clustered and genetically different from *Cx. perexiguus* found in the Iberian Peninsula and Middle East. Interestingly, in this study, while some samples clustered with *Cx. univittatus* (BEN18MP028, BEN17MP011, GAU14MP063/070), one pool clustered with *Cx. perixiguus*. Though difficult to explain, this may be because this pool was originally from Marakele (MAR14MP222), which is already in the Middleveld, and hence may belong to an intermediate form [49,50]. However, pools of mosquitoes are not suitable for the analysis of possible hybrids. Further studies crossing individual mosquito specimens’ molecular and morphological data would be vital, particularly for the *Univittatus* group, to understand their evolutionary relationship and aid in their correct identification, a necessary precursor to the development and implementation of vector control strategies.

These findings provide important evidence of the presence of three alphavirus species (MIDV, SINV, and NDUV) in South Africa. These arboviruses are of public health importance in the northeastern provinces, where human and animal cases of MIDV and SINV have previously been detected. Also reported here is the first detection of NDUV in these areas since the 1960′s, which raises the question of whether NDUV is currently of some veterinary and public health importance. An investigation of febrile cases in humans and animals in the area may shed light on whether this virus represents a potential zoonotic agent. No CHIKV-positive pools were identified during the study period, although outbreaks have been reported in the past in Northern KwaZulu-Natal and eastern Limpopo Provinces. The trap type used here did not target *Ae. furcifer*, the mosquito species which were involved in CHIKV transmission in the past [9,10]. These species are present in the canopy of the trees, and we have collected mosquitoes at ground level, which could account for the low presence of *Ae. furcifer* [9,10]. The concentration of these arboviruses in infected mosquitoes in rural, conservation, and peri-urban areas may indicate that livestock, wildlife, and/or birds common to these areas may be important for the amplification of these viruses. In-depth studies of these viruses, including vector competence and reservoir hosts, will be important to understanding the dynamics of the transmission, molecular epidemiology, and the development of future vector control measures.

## Figures and Tables

**Table 1:** Mosquito species that tested positive for alphaviruses.

*Culicidae* Alphaviruses Detected	N Mosq. Assayed	Pools Positive/Pool Tested	Infection Rate *
Middelburg virus			
*Ae. dentatus* gr.	471	1/21	2.1
*Ae. durbanensis*	716	2/17	1.4
*An. coustani*	2205	1/62	0.5
*Cx. terzii*	54	1/12	18.5
*Cx. univittatus*	4061	1/105	0.2
Sindbis virus			
*Ae. durbanensis*	716	1/17	1.4
*Ae. Aerarius/tarsalis*	58	1/3	17.2
*Cx. zombaensis*	33	1/5	30.3
*Cx. annulioris*	165	1/15	6.1
*Cx. univittatus*	4061	5/105	1.2
*Cx. pipiens* s.l.	2617	2/76	0.8
*Cx. theileri*	2129	1/70	0.5
*Ma. uniformis*	2472	1/67	0.4
Ndumu virus			
*Ae. mcintoshi*	3653	1/17	0.3
*Cx. annulioris*	165	1/15	6.1

* Minimum Infection Rate: ([Number of positives/number of mosquitoes assayed] × 1000). Ae.: *Aedes*; An.: *Anopheles*; Cx.: *Culex*; Ma.: *Mansonia;* N: Number; Mosq.: Mosquitoes.

## Data Availability

Not applicable.

## Supplementary Materials

The following are available online at https://www.mdpi.com/article/10.3390/v15020414/s1, Figure S1: Phylogenetic of the E gene alphavirus-positive sequences identified in this study. (**A**) Phylogenetic tree of the positive sequences based on the 27 sequences and 344 bp of the glycoprotein E1 gene comparing Middelburg virus positive pools to other alphaviruses. The tree was constructed by employing the program MEGA 7, using the maximum likelihood method based on the Kimura 2-parameter model with 1000 bootstrap replicates. The tree with the highest log likelihood (−4642.43) is shown. Numbers on internal branches indicate bootstrap values. The samples that are part of this study are marked with a triangle shape. (**B**) Phylogenetic tree of the positive sequences based on the 29 sequences and 445 bp of the glycoprotein E2 gene of Sindbis positive specimens relative to other alphaviruses. The tree was constructed by employing the program MEGA 7, using the maximum likelihood method based on the Tamura 3-parameter model with 1000 bootstrap replicates. The tree with the highest log likelihood (−6218.03) is shown. Numbers on internal branches indicate bootstrap values. Samples that are part of this study are marked with a dot shape. (**C**) Phylogenetic tree of the positive sequences based on the 26 sequences and 386 bp of the glycoprotein E1 gene of NDUV positive specimens relative to other alphaviruses. The tree was constructed by employing the program MEGA 7, using the maximum likelihood method based on the Kimura-2 parameter model with 1000 bootstrap replicates. The tree with the highest log likelihood (−6222.32) is shown. Numbers on internal branches indicate bootstrap values. Samples which are part of this study are marked with a dot shape. Figure S2: Phylogenetic tree of the mosquito pool positive sequences of the COI gene. The tree was constructed based on 45 sequences and 517 bp by employing the program MEGA 7, using the maximum likelihood method based on the General Time Reversible model with 1000 bootstrap replicates. GenBank accession numbers are indicated. Numbers on internal branches indicate bootstrap values. Samples which are part of this study are marked with a triangle. Table S1: Table with PCR assay, corresponding primers and probes, sequences data, amplicon sizes, and genome position. Table S2: Information of the mosquito pools that tested positive for alphaviruses. Culicidae species, vector surveillance site, identification number of Culicidae homogenate pool that tested positive for alphaviruses, pool size, and accession numbers. Table S3: Pairwise—distance analysis of the SINV full genomes identified with the strains most similar to them (Kenya BONI 2013 strains). The P-distance was calculated using MEGA 7. The numbers in columns indicate the percentage (%) nucleotide similarity.
