# Supplementary material for: Alphaviruses Detected in Mosquitoes in the North-Eastern Regions of South Africa, 2014 to 2018
Source: Viruses. 2023 Feb 1;15(2):414. doi: 10.3390/v15020414 (PMC9965626; doi:10.3390/v15020414)
Supplement: Supplementary file 1 [file viruses-15-00414-s001.zip › viruses-2132609-supplementary.pdf]

Supplementary File

Table S1: Table with PCR assay, corresponding primers and probes, sequences data, amplicon sizes and genome position.

PCR	Primer Name	Orientation / channel/ dye	Sequence (5' – 3')	Target gene	Fragment size (nt)	Genome position
Alpha First	Alpha+1	Sense	GAYGCTTAYYTTGAYATGGTTGATGG	nsP4	481	6137-6618
	Alpha-1	Antisense	KYTCYTCIGTRTGYTTTGTTCCTGG			
Alpha Nested	Alpha +2	Sense	GIAAYTGYAAYGTIACICARAT		198	6315-6513
	Alpha -2	Antisense	GCRAAIARIGCIGCIGCYYTIGGICC			
Alpha Nested Specific	MNF	Sense	GCAGCCTTTTGTCCGTCYAA		347	6285-6632
	MNR	Antisense	GGCTTCAAGTCRTAGGTTT			
	SNF	Sense	GCAACCTTYTGCCCCGCYAA		347	6285-6632
	SNR	Antisense	GGGACCAAATTATRCGTCT			
	MINV Probe	560	GCTTTAAGAAGTACGCATGCAACA-VIC			6482-6504
	SINV Probe	530	ATGACGAGTATTGGGAGGAGTTTG-FAM			6503-6525
MIDV E1	MID10475	Sense	GGTGCACGTTCCATATACCC	E	350	
	MID11045	Antisense	TCCCAATAGCAATCACCACA			
	MID10543	Sense	TGAACCACAAGGCTCCTTTC			
	MID10911	Antisense	CACTTTGCTGTGCAAGTGGT			
	SIN9106EF	Sense	ATCAAGATYAGCACCTCAGGACC		616	9106-9722
SINV E2	SIN9700ER	Antisense	TCTAATTGGAGGCTGATGTGTTT			
	SINV A1	Sense	AAAGGATACTTTCTCCTCGC3		526	9148-9674
	SINV A2	Antisense	TGGGCAACAGGGACCATGCA			
NUDV E1	ND 124 F	Sense	CACCCTAAAAGTGACGTT			124-141
	ND 632 R	Antisense	ATTGCAGATGGGATACCG			615-632

Table S2: *Culicidae* specie, vector surveillance site, identification number of *Culicidae* homogenate pool that tested positive for alphaviruses, pool size and accession numbers.

Culicidae Species	Site	Pool ID	Pool Size	Virus	Accession Number	COI Accession Number
Cx. terzii	Kyalami	KYA18MP050	1	MIDV	MT553110 (Ns4)	NA
Cx. univittatus	Benoni	BEN18MP028	20	MIDV	MT553109 (Ns4)	MW520835
Ae. dentatus gr	Benoni	BEN17MP026	36	MIDV	MT553113 (Ns4), MZ508482 (E1)	MW538030
An. coustani	Mnisi	KRU16MP021	9	MIDV	MT553112 (Ns4)	MW532836
Ae. durbanensis	Jozini	KZN18MP346	46	MIDV	NA < 200 bp (Ns4), MZ508481 (E1)	MW538028

Ae. durbanensis	Jozini	KZN18MP342	50	MIDV	MT553111 (Ns4), MZ508483 (E1)	MT877605
Cx. univittatus	Marakele	MAR14MP222*	51	SINV	MZ508493 (Ns4) / OL802949	MW520827
Cx. univittatus	Boschkop	GAU14MP063	51	SINV	MZ508489 (Ns4)	MW520833
Cx. univittatus	Boschkop	GAU14MP070*	50	SINV	MZ508486 (Ns4) / OL802945	MW520832
Cx. pipiens s. l.	Boschkop	GAU14MP018	31	SINV	MZ508487 (Ns4)	MW520834
Cx. univittatus	Kyalami	KYA14MP134*	55	SINV	MZ508492 (Ns4) / OL802947	NA
Cx. pipiens s.l.	Kyalami	KYA14MP133*	47	SINV	MZ508491 (Ns4) / OL802946	MW520830
Cx. zombaensis	Kyalami	KYA18MP048	4	SINV	MZ508490 (Ns4)	MW520829
Cx. theileri	Kyalami	KYA18MP054	16	SINV	MZ508487 (Ns4)	MW520828
Cx. annulioris	KNP	KNP17MP723	40	SINV	MZ508484 (Ns4)	MW520831
Ae. tarsalis/aerarius	KNP	KNP17MP727	50	SINV	NA < 200 bp	MW538033
Cx. univittatus	Benoni	BEN17MP011	50	SINV	MZ508485 (Ns4)	MW520836
Ma. uniformis	Mnisi	KRU17MP427	50	SINV	MZ508494 (Ns4)	NA
Ae. durbanensis	Jozini	KZN18MP345	50	SINV	NA < 200 bp	MW538029
Cx. annulioris	Lapalala	LAP18MP234	3	NUDV	NA < 200 bp	NA
Ae. mcintoshi	Jozini	KZN18MP347	18	NUDV	NA < 200 bp	MW538032

NA: Not available due to sequence smaller than 200bp. These sequences are available upon request. KNP: Kruger National Park
* Positive pools and detected the virus on cell culture.

Table S3: Pairwise – distance analysis of the SINV full genomes identified with the strains most similar to them (Kenya BONI 2013 strains). The P-distance was calculated using MEGA 7. The numbers in columns indicate the percentage (%) nucleotide

Complete genomes identified in the study and closely related Kenya BONI strains	Percentage (%) nucleotide similarity				
MAR14MP222_S2_L001_R1_001_(Reads)_U38305.1 _consensus					
GAU14MP070_S4_L001_R1_001_(Reads)_U38305.1 _consensus	100				
KYA14MP133_S1_L001_R1_001_(Reads)_U38305.1 _consensus	99,9	99,9			
KYA14MP134_S3_L001_R1_001_(Reads)_U38305.1 _consensus	99,9	99,9	99,9		
KY616987.1_Sindbis_virus_isolate_BONI_566_KENYA _2013_complete_genome	98,3	98,3	98,3	98,3	
KY616985.1_Sindbis_virus_isolate_BONI_584_KENYA _2013_complete_genome	98	98	98	98	98,9

similarity.

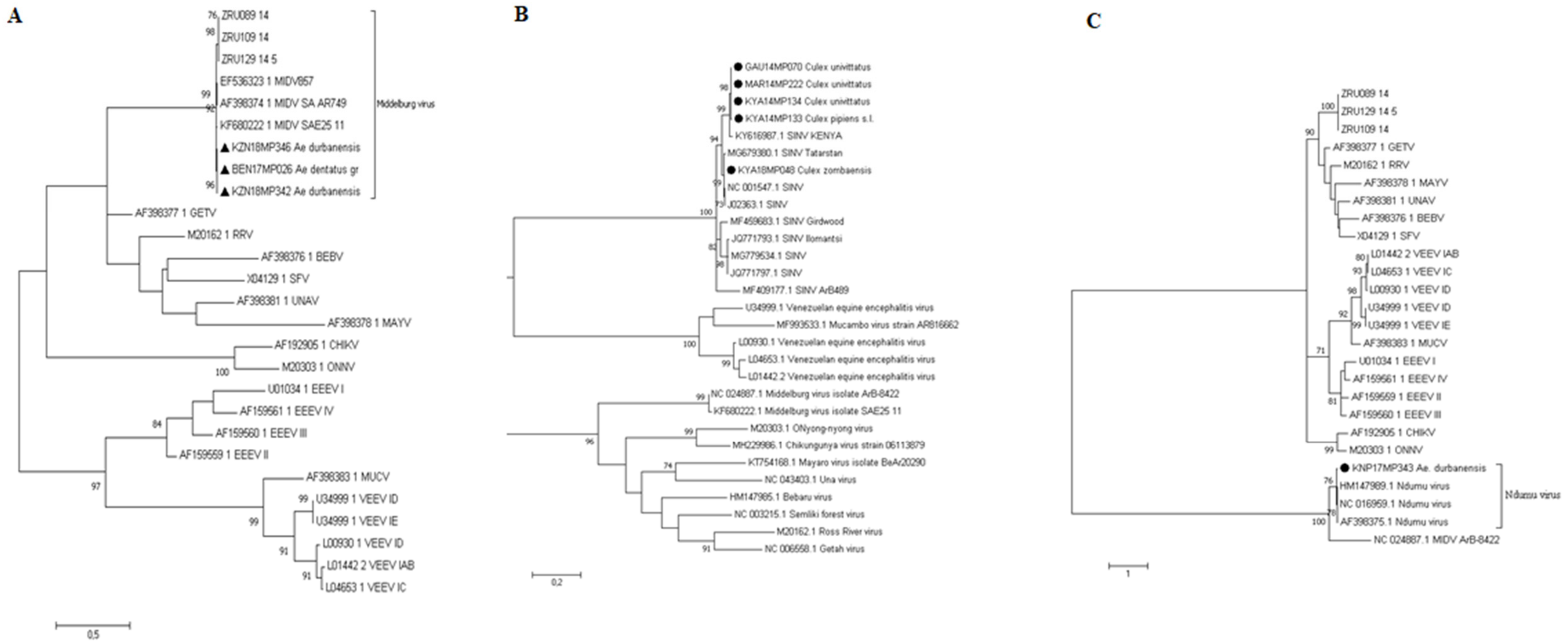

Figure S1: Phylogenetic of the E gene alphavirus-positive sequences identified in this study. (**A**) Phylogenetic tree of the positive sequences based on the 27 sequences and 344 bp of the glycoprotein E1 gene comparing Middelburg virus positive pools to other alphaviruses. The tree was constructed by employing the program MEGA 7, using the maximum likelihood method based on the Kimura 2-parameter model with 1000 bootstrap replicates. The tree with the highest log likelihood (−4642.43) is shown. Numbers on internal branches indicate bootstrap values. The samples that are part of this study are marked with a triangle shape. (**B**) Phylogenetic tree of the positive sequences based on the 29 sequences and 445 bp of the glycoprotein E2 gene of Sindbis positive specimens relative to other alphaviruses. The tree was constructed by employing the program MEGA 7, using the maximum likelihood method based on the Tamura 3- parameter model with 1000 bootstrap replicates. The tree with the highest log likelihood (−6218.03) is shown. Numbers on internal branches indicate bootstrap values. Samples that are part of this study are marked with a dot shape. (**C**) Phylogenetic tree of the positive sequences based on the 26 sequences and 386 bp of the glycoprotein E1 gene of NDUV positive specimens relative to other alphaviruses. The tree was constructed by employing the program MEGA 7, using the maximum likelihood method based on the Kimura-2 parameter model with 1000 bootstrap replicates. The tree with the highest log likelihood (−6222.32) is shown. Numbers on internal branches indicate bootstrap values. Samples which are part of this study are marked with a dot shape.

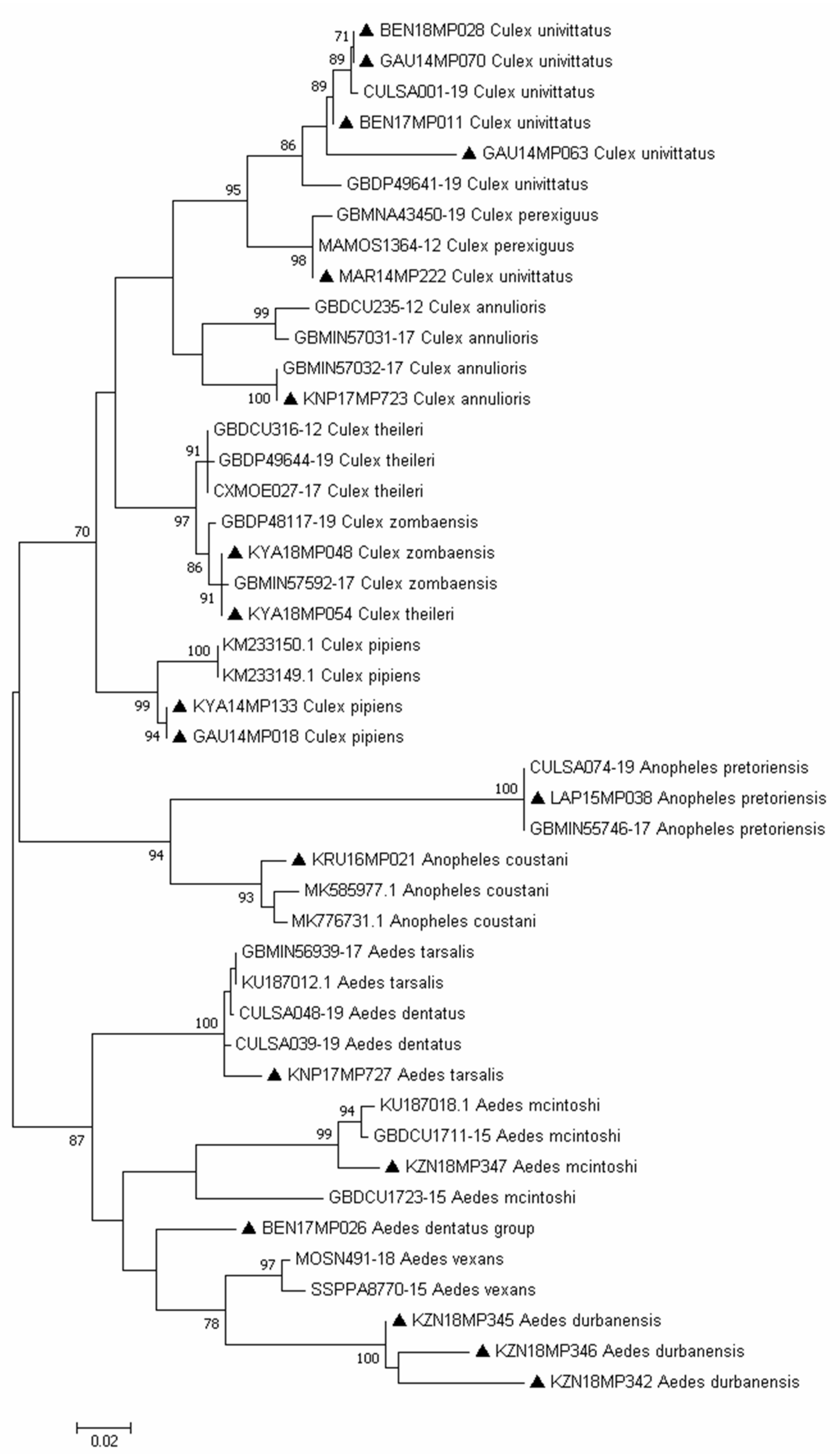

Figure S2: Phylogenetic tree of the mosquito pool positive sequences of the COI gene. The tree was constructed based on 45 sequences and 517 bp by employing the program MEGA 7, using the maximum likelihood method based on the General Time Reversible model with 1000 bootstrap replicates. GenBank accession numbers are indicated. Numbers on internal branches indicate bootstrap values. Samples which are part of this study are marked with a triangle.
